# Severe MUPS in a sick-listed population: a cross-sectional study on prevalence, recognition, psychiatric co-morbidity and impairment

**DOI:** 10.1186/1471-2458-9-440

**Published:** 2009-12-01

**Authors:** Rob Hoedeman, Boudien Krol, Nettie Blankenstein, Petra C Koopmans, Johan W Groothoff

**Affiliations:** 1 Department of Health Sciences, University Medical Center Groningen, University of Groningen, Groningen, The Netherlands; 2 Department of Science, ArboNed Occupational Health Services, Utrecht, The Netherlands; 3 Department of General Practice, EMGO Institute for Health and Care Research, VU Medical Center, Amsterdam, The Netherlands

## Abstract

**Background:**

Medically unexplained physical symptoms (MUPS) have a high prevalence in the general population and are associated with psychiatric morbidity. There are indications that MUPS are an important determinant of frequent and long-term disability.

The primary objective was to assess the prevalence of MUPS in sick-listed-employees and its associations with depressive disorders, anxiety disorders, health anxiety, distress and functional impairment. Secondary objectives were to investigate the classification of the occupational health physicians (OHPs), their opinions about the causes as well as the attributions of the employee.

**Methods:**

In a cross-sectional study of 489 sick-listed employees from 5 OHP group practices, MUPS, depressive disorders, anxiety disorders, health anxiety, distress and functional impairment were assessed with the Patient Health Questionnaire (PHQ), the Whitely Index (WI), the Four- Dimensional Symptom Questionnaire (4DSQ) and the Short-Form 36 Health Survey (SF-36).

We used a cut off score of 15 on the PHQ for the categorisation of severe MUPS.

The opinions of the OHPs were evaluated by means of a separate questionnaire with regard to the presence of employees physical symptoms, and the symptoms attributions, and the diagnoses of the OHPs.

**Results:**

Severe MUPS had a prevalence of 15.1% in this population of sick-listed employees. These employees had 4-6 times more depressive and anxiety disorders, and were more impaired. Female gender and PHQ-9 scores were determinants of severe MUPS.

Most of the time the OHPs diagnosed employees with severe MUPS as having a mental disorder. The employees attributed their physical symptoms in 66% to mental or to both mental and physical causes.

**Conclusion:**

The prevalence of severe MUPS is higher in long-term sick-listed employees than in the non-sick- listed working population and at least equals the prevalence in the general practice population.

Severe MUPS are associated with psychiatric morbidity and functional impairment and must therefore be specifically recognised as such. Validated questionnaires, such as the PHQ-15, are useful instruments in order to help OHPs to recognise severe MUPS.

## Background

Many studies have reported high prevalence rates for medically unexplained physical symptoms (MUPS), which, depending on the definition, vary from 10-24% in medical outpatients, primary care patients and the general population. These studies used the psychiatric definition of the somatoform disorders [1,2] and sub-threshold forms of somatoform disorders, such as abridged somatoform disorder (ASD) and multisomatoform disorder (MSD) [3-5], which can be seen as disorders with long-lasting and multiple MUPS. In these studies the patients with somatoform disorders also had a high prevalence of psychiatric co-morbidity, especially depression and anxiety disorder [1-5]. These patients also report functional impairments in all domains of health-related quality of life [6], and they have higher rates of unemployment [7].

MUPS, defined as physical symptoms which are not or insufficiently explained by a somatic disease, are also often associated with (work-)stress [8,9]. MUPS as symptoms must be distinguished from somatisation which describes a process in which the patient attributes the physical symptoms to a medical cause and seeks medical help, but no organic disease can be found [10].

In most studies patients were screened by psychiatrists, using a psychiatric interview as the golden standard, but in health care methods must be found that do not involve extensive and expensive screening by psychiatrists. In recent years studies have been performed using validated questionnaires to help doctors in primary care to diagnose somatoform disorders and MUPS. Examples are the Patient Health Questionnaire [5,11,12] (PHQ), the Symptom Checklist [13] (SCL-90) and the Four-Dimensional Symptom Questionnaire [14] (4DSQ). These questionnaires were also used to study the prevalence of MUPS and psychiatric disorders [11,15].

In the working, non-sick-listed population, low levels of MUPS and psychiatric co-morbidity have been reported [14], contrary to the findings in the sick-listed population. In various studies it has been found that sickness certified periods lasted longer and were more frequent in employees with no clear somatic diagnosis for their physical symptoms [16] and with multiple self-reported health symptoms [17-20].

These findings give rise to our hypothesis that in the long-term sick-listed population there is a higher prevalence of MUPS than in the working population, and that this is equal to or higher than the prevalence in the general population. The same applies to the levels of associated co-morbid psychiatry and functional impairment.

Sick leave is a major problem, that is associated with reduced well-being of the employee and high costs, due to reduced productivity and replacement [21] of the employee.

To our knowledge there has been no high quality study that has evaluated the recognition of MUPS and somatisation by occupational health physicians (OHPs), compared to validated instruments.

Curative care studies [22-24] show that primary care physicians have a low level of recognition of somatoform disorders (36-48%) according to the Diagnostic and Statistical Manual of Mental Disorders, Fourth Edition (DSM-IV) criteria. Patients with MUPS have symptoms of depression and anxiety in varying rates of prevalence, but their somatisation [24] and normalising attributional styles [23] make it difficult for physicians to recognise depressive and anxiety disorders.

Troublesome in this field is that different approaches (definitions of MUPS, somatoform disorders and somatisation) and different instruments (psychiatric interviews and questionnaires measuring different categories) are used. The different approaches make it difficult to compare studies regarding recognition and prevalence rates.

OHPs consider employees' symptoms in a biopsychosocial model as part of their management strategy. Nowadays Dutch OHPs are getting more used to the concept of somatisation as part of a four-dimensional approach, as advocated by Terluin et al. [25], of common mental disorders (CMD). This approach has been integrated in the Dutch guideline for OHP management of employees with CMD [26]. For most Dutch OHPs MUPS is an unknown concept, and is not included in the current guidelines. We hypothesised that when identifying psychosocial factors, usually OHPs would diagnose MUPS as stress-related mental disorders, but that they would also more diagnose somatisation as a secondary diagnosis.

The law in the Netherlands states that employees who report sick must be seen before the sixth week of sick leave (for the first consultation) by an OHP. The OHP establishes the diagnosis, the disabilities, and the prognosis for return to work. The maximum period of sickness certification is two years.

In the present paper the research questions are:

1. What is the prevalence of severe MUPS in sick-listed employees, seen by OHPs, and what are the associations with depression, anxiety disorders, distress and health anxiety, duration of sickness absence on the consultation day and functional impairment?

2. What are the OHPs' diagnoses, what are their opinions about the causes of severe MUPS, and what are the symptom attributions of the sick-listed employees with severe MUPS, compared to the symptom attributions of sick-listed employees with non-severe MUPS?

## Methods

### Design

The study was designed as a cross-sectional survey.

### Selection of employees

Sick-listed employees were included in the study from April 2006 until December 2007 by 43 OHPs, in 5 group practices, derived from two large occupational health services. The exclusion criteria were:

1) Insufficient mastery of the Dutch language.

2) Consultation by telephone.

### Selection of OHPs

We chose a mixture of group practices, providing services to large organisations (> 500 employees), medium-sized organisations (75-500 employees) and small organisations (< 75 employees) from different branches (Table 1).

**Table 1:** Study population

*OHP group practice*	***1***^*a*^	***2***^*b*^	***3***^*a*^	***4***^*a*^	***5***^*b*^
Employees (n)	85	87	108	98	111
OHPs (n)	13	7	6	8	9
Size organizations (nr of employees)	> 500	> 500	< 75	< 75; 75-500	> 500
Main branches	public services, education and health services	government	all types	all types	Public and financial services, local government
Area	Urban	Urban	Rural	Mixed	Urban

a = Arboned; b = Achmea Vitale

### Study size

In this study population we assumed an equal or higher prevalence, of at least 10%, than in the primary care population, with a worst acceptable rate being 7%. With a 95% confidence interval, this implies that the sample should consist of at least 384 employees.

We decided to include at least 40 OHPs, performing 6 sessions, of at least 4 consultations of employees fulfilling the inclusion criteria. This would result in 960 eligible employees. Assuming a maximum non-response of 50% from the employees and a maximum non-response of 15% from the OHPs, at least 408 employees were expected to be included in the study.

### Data-collection

Over a period of six weeks the OHPs were asked to select a 4-hour consultation session every week on the same day (i.e. each Monday from 8-12 a.m.). The practice assistants in the administrative section of the occupational health service were instructed to invite all sick-listed employees, who had an appointment for this session to participate in the study. These employees received the research questionnaires one week before the actual consultation, or later if they received the invitation after that time. They were also requested to give informed consent. The OHPs were not involved in the selection of the patients.

The questionnaires were collected on the day of the consultation by the researcher (RH), just before the consultation with the OHP. After the consultation the OHP filled in the questionnaire about the presence of physical symptoms, the diagnosis, the employee's symptom attributions, and the OHP's own opinion about the causes of the symptoms.

Employees who had forgotten to bring their questionnaire were asked to send their questionnaire to the researcher within one week, otherwise they would be considered as non-responders, and no reminders were sent.

### Measures

#### a) Questionnaires for the employee

The employees were asked to answer questions about their socio-demographic variables, MUPS, depression, anxiety, distress, health anxiety and functional impairment.

##### MUPS

The Patient Health Questionnaire (PHQ) [5,12,27] assesses MUPS and symptoms of depression, anxiety, distress, eating disorder and alcohol abuse. The PHQ-15 assesses MUPS and rates the extent to which the patient has been bothered during the past four weeks (score 0-2; not at all bothered to bothered a lot) by 15 common somatic symptoms (e.g. fatigue, dizziness, headache) that rarely have organic explanations.

The total PHQ-15 score range from 0-28 for men and 0-30 for women. For the diagnosis of a somatoform disorder a clinician's assessment is required, but high correlation has been reported between the PHQ-15 score and clinician-rated symptoms of somatoform disorder [28]. Kroenke indicated cut-off scores of 5, 10 and 15 for mild, moderate and severe MUPS. The cut-off point of 15 (PHQ-15 ≥ 15) is comparable with clinically representative samples of MUPS [5,27]. Patients with a PHQ score < 15 are described as patients with non-severe MUPS, indicating they have moderate, mild or no MUPS. In this study we compare employees with severe MUPS with employees with non-severe MUPS. Kroenke found in a primary care population a prevalence of 9% and in a secondary care population a prevalence of 10% of severe MUPS [5].

The internal consistency of the PHQ-15 is satisfactory (Cronbach's a = 0.80) [12,28]. The test-retest reliability in a high risk primary care population was moderate with 0.60 [12]. Although limited research has been done, these figures indicate a valid and moderately reliable questionnaire for detection of patients at risk for somatoform disorders [5,12,27,28].

We also used the Four-Dimensional Symptom Questionnaire (4DSQ) to measure MUPS. This Dutch self-report questionnaire [14,25] assesses the dimensions of distress, MUPS, anxiety and depression. The questionnaire is internally consistent, with Cronbach's alpha coefficients from .79 (anxiety), to .90 (distress) assessed in a working population [14], without sick-listed employees (personal information). The Cronbach's alpha coefficient was 0.80 for MUPS, assessed in a working population. Compared to the diagnosis of General Practitioners (GPs) of somatisation, the Area Under the Curve (AUC) was 0.62 [25]. We used the MUPS subscale of the 4DSQ, in addition to the PHQ-15, to allow comparison of our findings with other studies among employees, since to our knowledge there are no studies which used the PHQ-15 in a working population.

##### Depression

The PHQ-9 was used to assess symptoms of depression [29,30]. The rating is comparable with the PHQ-15. Two questions (feeling tired and having trouble sleeping) in the PHQ-9 are also included in the PHQ-15. Although this makes the PHQ-9 score less independent of the PHQ-15 score, high construct validity and strong associations with clinical variables in the general population are found [31]. Compared to the Hospital Anxiety Depression Scale (HADS) the PHQ-9 categories a higher proportion with moderate or severe depression [32].

Total PHQ-9 score ranges from 0-27, with a cut-off point of 15 (PHQ-9 ≥ 15) for severe levels of depression. Algorithms are applied to indicate major depressive disorder or any depressive disorder (excluding the other diagnosis).

##### Anxiety disorders

The PHQ anxiety subscale contains a module for the assessment of the symptoms of panic disorder and a module for symptoms of other anxiety disorders [27]. Algorithms are applied to diagnose panic disorder and other anxiety disorders. The algorithm for the panic disorder module has been more validated [27,33] than the algorithm for other anxiety disorders, and has a sensitivity of 75% and a specificity of 96% for diagnosing panic disorder [33].

##### Distress

We used the Four-Dimensional Symptom Questionnaire (4DSQ) to measure symptoms of distress. The distress subscale is associated with (job-) stressors and indicators of strain. The total score for the 16 distress symptoms range from 0-32, with a cut-off point of 20 for severe levels of distress [14,25].

##### Health anxiety

The Whitely index (WI) was used to measure health anxiety. This 14-item self-report questionnaire with yes/no questions was designed to assess health anxiety [10,34].

##### Functional impairment

The Dutch translation of the Short Form Health Survey (SF-36) was used [35] to measure levels of functioning, perceived disability and health related quality of life. The SF-36 has high validity [36], and measures eight aspects of health-related quality of life (physical functioning, role functioning physical, bodily pain, general health perceptions, vitality, social functioning, role functioning mental and mental health), with higher scores indicating higher levels of functioning and well-being.

#### b) Questionnaire for the OHP

The questionnaire for the OHP contained questions about:

- the presence of physical complaints (yes/no), as reported by the employee

- the symptom attribution as reported by the employee (somatic, mental, or both causes, physiological or not clear)

- the OHP's diagnosis, with one classification according to the CAS classification (classification for occupational health and social insurance), derived from the IDH classification (international standard for diagnostic classification).

- the opinion of the OHP about the causes of the symptoms (somatic disorder, distress, psychiatric disorder, hypochondriasis or somatisation). Example of a question is: 'Do you think that the physical symptoms of the employee are explained by distress?' The OHP was asked whether one or more explanations were present. The questions had a 4-point answering scale: completely, partly, (almost) not and unclear

The OHPs also filled in a questionnaire with regard to their personal socio-demographic status.

#### c) Registrations

Data on sick report and return to work (RTW) were collected from the computerized registration of the two participating occupational health services.

### Data analyses and statistics

A non-response analysis was performed on age, gender, level of educational, ethnicity and duration of sick leave on the day of the consultation.

The cut-off point for the MUPS score (PHQ-15) was set at 15. The data were dichotomized revealing a PHQ ≥ 15 group (the PHQ 15+ group) and a PHQ <15 group (the PHQ 15- group). Chi-square tests were performed for categorical variables, and Fischer's Exact Tests were performed when more than 20% of the expected cell frequencies were less than 5. Chi-square tests for trend for ordinal variables were performed. Independent Students' *t *tests were performed for continuous and Mann-Whitney U tests for non-parametric distributions.

The SF-36 scores were compared for the PHQ 15+ and PHQ 15- group with respectively no psychiatric morbidity, one psychiatric disorder, mixed psychiatric morbidity (one depressive and one anxiety disorder) and three psychiatric disorders (major or other depressive disorder, panic disorder and other anxiety disorder). We performed a multivariate (MANOVA) and univariate analyses with the 8 SF-36 levels as dependent variables.

Psychiatric morbidity (0, 1, 2 and 3 disorders) and the PHQ score (15+ and 15- group) were the independent variables and we adjusted for gender, age and ethnicity. Also the interaction between psychiatric morbidity and the PHQ score was tested. If influence of the psychiatric morbidity was found we performed posthoc analyses. We also reported R^2^, which estimates the proportion of explained variance.

To identify the determinants of the PHQ score a logistic regression model was conducted. In this model with the PHQ score as the dependent variable, based on the literature [1-7]. As independent variables were chosen: gender, age, ethnicity, group practice, attribution of the employee, PHQ-9, WI, distress, panic disorder and anxiety disorder.

The Hosmer and Lemeshow Goodness-of-Fit test was applied, which divides subjects into ten equally sized groups, based on predicted probabilities and computes a Chi-square from observed and expected frequencies. If the p-value of the Hosmer and Lemeshow Goodness-of-Fit was .05 or less we rejected the zero hypothesis that there is no difference between the observed and the predicted values of the dependent variable. A high significance level implies a good fit of the model. We also report the Nagelkerke R^2^, which estimates the proportion of explained variance in a logistic regression model.

All analyses were performed in SPSS for Windows 15.0.

Ethical approval was obtained form the Medical Ethics Committee of the University Medical Center in Groningen, who informed us that ethical clearance was not required because only self-report questionnaires were used and the study reports at group level.

## Results

The eligible study population consisted of 812 sick-listedemployees; 489 employees completed the questionnaires (response rate: 60.2%).

**Figure 1:**
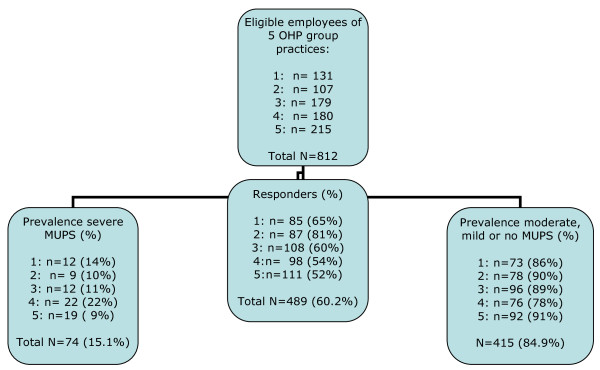
**Flow chart regarding response and prevalence in group practices and total Sample**.

Non-responders were comparable to responders with regard to gender and duration of sick leave on the day of the consultation, but the non-responders were younger (mean 41.7 years vs. 44.6 years, p = 0.001). We found a prevalence rate of 15.1% (n = 74) for severe MUPS. See Figure 1.

The characteristics of the employees in the PHQ 15+ group and the PHQ 15- group are presented in Table 2. In the PHQ 15+ group females were over-represented (p = 0.008). The employees in the PHQ 15+ group were about 4 to 6 times more likely to have symptoms of a major depressive disorder, panic disorder and other anxiety disorders, compared to the employees in the PHQ 15- group.

**Table 2:** Severe MUPS and univariate associations with socio-demographic variables, psychiatric morbidity, health anxiety, distress, OHP diagnosis, attribution employee and duration of sickness absence on consultation day

	*PHQ 15+* *N = 74*	*PHQ 15-* *N = 415*	***p-value***^*a*^
Female %	73.0	56.6	0.008
Age mean (SD)	42.5 (9.2)	45.0 (10.0)	0.040^c^
Education % High/average/low	20.0/55.7/24.3	35.5/44.4/19.6	0.031^b^
Autochtone %	75.3	88.6	0.003
Married/living together/alone %	46.6/11.0/42.5	57.7/14.7/27.6	0.038
PHQ-15 mean (SD)	19.2 (2.6)	8.2 (3.9)	<0.001^d^
Major depressive disorder %	64.9	14.7	<0.001
Other depressive disorder %	10.8	13.5	0.528
PHQ-9 mean (SD)	16.5 (5.4)	7.2 (5.6)	<0.001^d^
Panic disorder %	21.6	3.6	<0.001
Other anxiety disorder %	44.6	10.6	<0.001
4DSQ severe MUPS mean (SD)	22.6 (5.5)	9.4 (6.0)	<0.001^d^
Whitely Index (health anxiety) mean (SD)	7.0 (2.9)	4.1 (2.9)	<0.001^d^
Physical symptoms %	94.5	88.6	0.128
			
OHP diagnosis*(%)			
Mental disorder	62.2	39.8	<0.001
Musculoskeletal disorder	10.8	29.6	<0.001
Other disorder	27.0	30.6	0.537
			
Attribution** *employee to*^a ^(%): Physical causes	30.4	54.3	p < 0.001
Mental causes	26.1	15.4	p = 0.031
Both causes	40.6	23.7	p = 0.004
Physiological causes	1.4	6.1	p = 0.118
Do not know	1.4	0.6	P = 0.407
			
Duration of sick leave (days) on day of consultation			
Median	135	121	0.248^d^
Mean (SD)	238.7 (283.2)	176.1 (190.4)	

* CAS-classification of OHP

** For employees with physical complaints

^a ^X^2 ^test; ^b ^X^2 ^trend test;^c ^Independent Students' t test;^d ^Mann-Whitney U test

Levels of health anxiety and distress were higher in the PHQ 15+ group. The mean levels of MUPS in the PHQ 15+ group were 19.2 (SD 2.6) as measured with the PHQ and measured with the 4 DSQ 22.6 (SD 5.5), for depression (PHQ-9) 16.5 (SD 5.4) and for distress (4DSQ) 22.6 (SD 5.5).

In the PHQ 15+ group mental diagnoses were more frequently diagnosed and musculoskeletal disorders less than in the PHQ 15- group. Posthoc analysis showed that adjustment disorders were more diagnosed than psychiatric disorders in the PHQ 15+ group.

On average the consultation took place after a median period of sick leave of 123 days, and a mean period of sick leave of 185 days (SD 207.5), with a trend that employees in the PHQ 15+ were sick-listed for a longer period (Table 2).

Quality of life and levels of functioning of the employees with severe MUPS were lower than that of the employees with moderate, mild or non MUPS in all domains of the SF-36. The results are presented in Table 3. In the PHQ 15+ group without psychiatric morbidity, levels of functioning especially in the domains of general health perceptions (p = 0.001), vitality (p = 0.001) and mental role functioning (p = 0.002) were lower than in the PHQ 15- group without psychiatric morbidity.

**Table 3:** Associations of severe MUPS and psychiatric morbidity with functional limitations

*SF-36 scale mean (SD)*	*Total population*	*PHQ 15+*	*PHQ 15-*
Study population	n = 489	n = 74	n = 412
Without a depressive or anxiety disorder	n = 298	n = 13	n = 285
With only 1 depressive *or *1 anxiety disorder	n = 116	n = 26	n = 90
With only 1 depressive *and *1 anxiety disorder	n = 62	n = 26	n = 36
With 3 mental disorders	n = 13	n = 9	n = 4
SF physical functioning	69.0 (24.6)	55.3 (24.0)	71.6 (23.8)
	71.5 (23.4)	72.7 (16.8)	71.5 (23.7)
	67.0 (26.2)	47.7 (23.0)	72.7 (24.4)
	62.6 (24.8)	54.4 (22.7)	68.7 (24.7)
	61.5 (27.4)	55.0 (29.8)	76.3 (14.9)
SF role functioning physical	26.1 (36.2)	12.7 (28.0)	28.4 (37.0)
	30.1 (37.7)	14.6 (29.1)	30.7 (37.9)
	20.4 (32.0)	4.8 (14.2)	25.0 (34.3)
	18.3 (33.8)	18.0 (34.2)	18.6 (33.9)
	20.8 (39.6)	18.8 (37.2)	25.0 (50.0)
SF bodily pain	53.3 (27.7)	36.6 (22.8)	56.3 (27.5)
	57.4 (28.1)	47.9 (15.7)	57.8 (28.5)
	48.9 (25.7)	30.5 (19.2)	54.2 (24.9)
	44.0 (25.0)	40.0 (26.6)	46.9 (23.8)
	42.4 (31.0)	27.9 (23.3)	75.0 (19.0)
SF general health perceptions	54.2 (20.2)	38.2 (15.0)	57.0 (19.7)
	60.1 (18.9)	46.5 (12.3)	60.7 (18.9)
	47.1 (19.6)	35.8 (12.1)	50.4 (20.1)
	43.7 (17.4)	39.8 (17.5)	46.5 (17.0)
	31.5 (11.8)	28.9 (12.9)	37.5 (6.5)
SF vitality	44.6 (22.2)	26.6 (16.8)	47.9 (21.6)
	55.2 (19.5)	38.1 (14.7)	56.0 (19.3)
	31.8 (14.8)	34.0 (15.4)	31.2 (14.7)
	22.6 (13.8)	15.6 (11.3)	27.6 (13.3)
	21.9 (14.4)	20.6 (17.0)	25.0 (5.8)
SF social functioning	53.6 (26.8)	36.8 (25.2)	56.6 (26.0)
	63.8 (24.3)	57.7 (24.8)	64.1 (24.3)
	41.3 (22.9)	40.4 (23.8)	41.6 (22.8)
	33.7 (19.8)	29.3 (20.6)	36.8 (18.9)
	23.1 (23.3)	18.1 (20.8)	34.4 (27.7)
SF role functioning mental	52.6 (44.9)	19.3 (36.3)	58.3 (43.7)
	71.3 (39.5)	38.9 (39.8)	72.7 (38.9)
	30.3 (40.3)	24.0 (41.4)	32.2 (40.1)
	10.6 (23.4)	4.2 (20.4)	14.8 (24.5)
	19.4 (33.2)	20.8 (39.6)	16.7 (19.2)
SF mental health	60.7 (23.2)	41.1 (22.2)	64.2 (21.6)
	73.5 (16.2)	65.2 (13.0)	73.8 (16.3)
	47.7 (16.4)	51.4 (17.2)	46.6 (16.1)
	31.7 (13.7)	25.2 (11.7)	36.3 (13.4)
	23.4 (17.3)	22.2 (19.7)	26.0 (12.4)

Multivariate analysis showed that psychiatric co-morbidity had an overall effect of reduced functioning (p < 0.001) and that severe MUPS (PHQ 15+) also resulted in reduced functioning (p < 0.001); gender was the only confounder (p = 0.028). The effect of psychiatric co-morbidity was higher on reducing functioning in the PHQ 15+ group than in the PHQ 15-group (interaction effect: p = 0.016). The R^2 ^for our model was 0.551.

Posthoc analysis showed that employees with a PHQ 15+ score with one psychiatric disorder had a higher level of impairment in most domains than employees with a PHQ 15- score with one psychiatric disorder. In the PHQ 15+ group, more psychiatric disorders did not contribute substantially to the levels of impairment, except for vitality, social functioning and mental health.

In the PHQ 15- group, the pattern was different: general health perceptions and the mental domains of reduced functioning correlated with the number of co-morbid disorders. Physical functioning was not influenced by psychiatric morbidity.

Of the sick-listed employees 89.5% told the OHP that they had physical symptoms. The causes to which the employees attributed the physical complaints are also shown in Table 2. In the PHQ 15+ group the number of physical complaints was comparable to that in the PHQ 15- group. In the severe MUPS group, attribution to physical causes was lower and attribution to mental causes was higher.

The OHP categorisation of somatisation was low: 35.9% of the employees of the PHQ 15+ group were partly or completely indicate as somatisers. In contrast 27.9% of the employees in the PHQ 15- group were partly or completely indicated as somatisers. If both MUPS and somatisation would have been one concept (which they are not), this would have resulted in a sensitivity of 0.36 (95% CI 0.25-0.49) and a specificity of 0.72 (0.67-0.77).

Posthoc analysis (see Table 4) showed that attribution of the OHP to somatic causes and distress was influenced by the presence of psychiatric co-morbidity. More than one psychiatric co-morbid disorder added to the OHP's opinion of somatic causes being less likely and distress more likely in the PHQ 15+ and PHQ 15- group and psychiatric causes more likely in the PHQ 15- group. This pattern was not found with regard to the causes of somatisation and hypochondriasis in both groups, and psychiatric causes in the PHQ 15+ group.

**Table 4:** Associations of severe MUPS and psychiatric morbidity with attribution of physical symptoms by employees and OHPs

		PHQ 15+	PHQ 15+
Study population*		n = 69	n = 365
Without a depressive or anxiety disorder	= 0	n = 13	n = 260
With 1 depressive or 1 anxiety disorder	= 1	n = 24	n = 74
With 2 or 3 mental disorders	= 2-3	n = 32	n = 31
*Attribution employee to *(%):			
Physical causes	0	23.1	10.5
	1	8.3	24.3
	2-3	40.6	35.5
Mental causes	0	46.2	64.0
	1	50.0	35.1
	2-3	9.4	19.4
Both causes	0	23.1	19.0
	1	37.5	32.4
	2-3	50.0	41.9
Physiological causes	0	0.0	6.2
	1	4.2	6.8
	2-3	0.0	3.2
Employee does not know	0	7.7	0.4
	1	0.0	1.4
	2-3	0.0	0.0
			
*OHP attribution of symptoms completely/partly to *(%):			
Somatic causes	0	46.2/30.8	59.0/16.3
	1	37.5/29.2	23.3/34.2
	2-3	6.7/20.0	16.1/25.8
Psychiatric causes	0	23.1/15.4	4.8/9.6
	1	16.7/25.0	19.2/24.7
	2-3	33.0/33.0	25.8/38.7
Distress	0	38.5/30.8	20.7/16.3
	1	12.5/29.2	28.8/46.6
	2-3	55.2/20.7	41.9/25.8
Health anxiety	0	15.4/23.1	1.2/15.5
	1	4.2/20.8	8.2/13.7
	2-3	6.7/46.7	3.2/38.7
Somatisation	0	0.0/38.5	4.8/18.7
	1	8.3/25.0	8.2/27.4
	2-3	6.7/30.0	9.7/35.5

*) For sick-listed employees with physical complaints

With regard to the opinion of the employee more than one psychiatric co-morbid disorder added to the opinion of mental causes being less likely, physical and both causes more likely in the PHQ 15+ and 15- group.

The logistic regression model with PHQ as dependent variable showed that gender of the employee was a determinant, as known from literature. After correction for socio-demographic variables and variation among the population from the different group practices, the score for depressive symptoms (PHQ-9) also appeared to be a determinant, in contrast to the other independent variables. The total explained variance, according to Nagelkerke R^2^, was 0.55. The results are shown in Table 5.

**Table 5:** Logistic regression model for PHQ-15 score as dependent variable^a^

Independent variables	OR	95% CI lower	95% CI upper	**Signif**.
Female gender	3.51	1.41	8.78	**0.007**
Age (per year)	0.98	0.94	1.02	0.405
Autochthons^B^	0.44	0.16	1.27	0.129
Group practice				0.332
Group practice 1	0.53	0.11	2.45	0.413
Group practice 2	0.70	0.21	2.34	0.559
Group practice 3	0.44	0.14	1.45	0.178
Group practice 4	1.43	0.52	3.94	0.493
PHQ-9	1.20	1.08	1.33	**0.001**
Whitely Index	1.12	0.97	1.28	0.116
Stress^c^	1.63	0.94	2.82	0.082
PHQ panic disorder	2.39	0.68	8.46	0.176
PHQ other anxiety disorders	1.20	0.46	3.12	0.709
Attribution employee to:				0.658
- mental causes	0.62	0.19	1.98	0.418
- physical and mental causes	0.66	0.24	1.80	0.413
- physiological causes	0.28	0.03	2.98	0.290

a) For sick-listed employees with physical complaints; b) born in the Netherlands; c) per 8 points on the 4DSQ.

Hosmer & Lemeshow test: N = 386 chi2 = 5.0 df = 8 p = 0.756

Nagelkerke R^2 ^= 0.55

## Discussion

### Prevalence of severe MUPS in sick-listed employees

Our data show that severe MUPS, measured with the PHQ-15, have a prevalence of 15.1% in the sick-listed population. Compared to the findings of Kroenke [5] in a primary care population and medical outpatients as assessed by the PHQ-15, the prevalence in our sample of sick-listed employees was higher. In the primary care population [2] and the general population [3] prevalences of 16% respectively 22% for somatoform disorders have been found. In the primary care population a prevalence of 16% was found for somatoform disorders with undifferentiated somatoform disorder (prevalence 13%) as the most frequent disorder, indicating that patients with this disorder were bothered by MUPS for longer than 6 months. Although we only studied employees who indicated that they had been bothered by severe multiple MUPS for 4 weeks, and we performed no medical examinations, the prevalence rates may be compared, assuming that the long-term sick-listed population had severe MUPS for longer than 4 weeks. In the study among the general population [3] the diagnosis of ASD was used, defined as 4-6 periods of MUPS in a life-long period. Therefore we can make no comparisons because information about the life-long medical history of the employees in our study was lacking. The mean MUPS score (11.4 on the 4 DSQ, SD 7.5) is substantially higher than the score found by Terluin (3.7 on the 4 DSQ, SD 4.1) in a working, non-sick-listed population [25]. On the one hand it should be taken into account that the median duration of sick leave was 123 days and a mean duration of 185 days (SD 207.5), indicating a selection of employees with a difficult RTW process. On the other hand, MUPS were not associated with the duration of sick leave on the day of the consultation. This may indicate that MUPS are already a serious problem when employees consult the OHP because they are sick-listed.

The prevalence we found is based on a sample of employees, 89% of whom reported one or more physical complaints. This is comparable to the rate of 86% found in a general practice population by Van der Windt [37].

Our conclusions are that the prevalence of severe MUPS in the sick-listed population is substantially higher than in the working population, that it is at least equal to the prevalence in the general practice population and that it might be even higher.

### Associations with psychiatric morbidity, health anxiety and distress

MUPS are associated with high levels of major depression, panic disorder and other anxiety disorders. This is in line with the findings in the general population [1-3], but the percentage of employees with severe MUPS without psychiatric morbidity is lower among sick-listed employees than in the general population.

The algorithms used in the PHQ-9 indicate that the diagnosis of major depressive disorder excludes the diagnosis of other depressive disorders, which explains the low prevalence of the latter in our findings. We also found that employees with severe MUPS experienced high levels of health anxiety and distress, but the cause of the distress cannot be derived from our data.

The logistic regression model showed that distress and health anxiety were not determinants of the PHQ-15 score, in contrast to the depression score. Patients often present the physical symptoms of their depression, and our data confirm this overlap of depression and MUPS in sick-listed employees. The OHPs recognised a high level of mental disorders, although not very specific because they more often diagnosed adjustment disorders than depressive disorders and anxiety disorders. The attribution of the employee could have influenced these diagnoses.

### Associations of severe MUPS and psychiatric co-morbidity with functional limitations

Compared to the findings of Kroenke [6] in a general practice population, severe MUPS had more impact on functioning in sick-listed employees: the SF-36 scores in our study were lower for all domains. Psychiatric morbidity adds to this effect, in line with the findings of Van de Waal et al. [2].

In sick-listed employees it also seems that severe MUPS, especially when accompanied by a psychiatric disorder, impairs functioning to such an extent that multiple psychiatric co-morbidity has no additional effect on most domains of functioning. This is in line with the findings of Barsky [15] i.e. that in primary care patients severe MUPS resulted in increased medical consumption and costs, but there was no further increase due to psychiatric morbidity. These findings imply that recognising MUPS and psychiatric co-morbidity are important for the OHPs, because of the impact on functioning.

### Recognition by the OHP

The OHPs' recognition of somatisation, compared to the PHQ-15 score, was low. The OHPs did not consider this as a primary or secondary cause of the symptoms. Because they diagnosed most often a mental disorder, with a stress-related cause, our conclusion is that for the OHPs the concept of somatisation is not related to severe MUPS. Therefore, our hypothesis that OHPs would recognise employees with severe MUPS more often as somatisers was not confirmed.

We did not ask the OHPs if they diagnosed MUPS, but in employees with severe MUPS they less often found a somatic explanation for employees with severe MUPS than in employees with moderate, mild or no MUPS. Hence our recommendation is that the concept of MUPS should be introduced to OHPs in guidelines and training.

Van Ravestijn et al. [12] found a low predictive value of the PHQ-15, which indicates that in the PHQ 15+ group the employees often had other diagnoses as depression and anxiety disorder. Indeed, our data pointed out that OHPs often diagnosed a mental disorder in the PHQ 15+ group.

In our sample, higher scores for depression contributed to a higher MUPS score. This psychiatric morbidity was not sufficiently recognised by the OHP, but it is well known in general practice [2], and is also in line with the findings of Clarke and Kessler i.e. that somatisation [24] and normalising attributions [23] hamper the recognition of depression and anxiety.

A point of interest is that many employees with severe MUPS did not attribute their physical symptoms to somatic causes only. This contributes to the growing evidence that 'the somatic pathway' in the doctor-patient communication is not mainly due to the somatic attributions of patients [38], and that the concept of somatisation should be applied on another level. Our data further show that more than one psychiatric co-morbid disorder influences the opinion of the employee with severe MUPS by finding only mental causes less likely and physical causes more likely, but also finding both causes more likely. When more than one psychiatric disorder is present the OHP finds somatic causes less likely and distress a more likely cause, but still not labels this as somatisation. Further conclusion is that in case of sick-listed employees with severe MUPS and more psychiatric co-morbid disorders there is a potential of difficulties between OHP and employee with regard to management and communication, because of the different opinions about the causes of the symptoms.

Somatisation is a specific factor, related to psychiatric co-morbidity, to the cognitions and coping styles of patients [38,39] and to factors in the physician-patient relationship [38-40]. Patients with MUPS who attribute their symptoms solely to a physical cause ('complete somatisers') have a worse prognosis than patients who do so partly or not at all [38-40]. Our results indicate that OHPs need more training to distinguish between MUPS, psychiatric co-morbidity and the employees attribution.

### Strengths and limitations

A strength of our study is that, to our knowledge, this is the first large scale study in which validated questionnaires have been used to assess severe MUPS and psychiatric morbidity in a sick-listed population. The participation of five different group practices located in urban and rural sites, and the participation of employees from small and large companies enhances the generalisability of the results. Furthermore, this is a cross-sectional survey in a population with moderate and long-term sickness absence, whereas other studies of sick-listed populations focused on diagnoses in employees with lasting impairment.

Another strong point is that we could compare the information gathered, by means of psychometrically validated questionnaires as reference test, to the diagnoses of the OHPs as well as to the opinions of the employees.

A limitation of a cross-sectional study, is that no conclusions can be drawn with regard to the underlying causes of severe MUPS. This is especially important with regard to the associations between severe MUPS on the one hand, and psychiatric co-morbidity, distress and health anxiety on the other hand.

Self-report questionnaires were used for our main outcomes, and no medical examination was performed to find somatic explanations for the multiple physical complaints. However, the questionnaires used were well validated, and there is sufficient evidence in the literature exists that a high number of somatic symptoms and PHQ levels *of *≥ 15 indicate that the symptoms should be considered as MUPS [5,28].

A response rate of 60.2% is only moderate. Nevertheless this is satisfying compared to response rates, varying from 30-60%, reported in other studies in the working population [41,42]. The non-responders were younger than the responders, which could have contributed to a somewhat higher prevalence rate in our sample, because the mean age of employees with severe MUPS tends to be higher [43]. There were no other indications of selection bias.

Finally, a limitation is that the OHPs were asked about their attribution of the symptoms to somatisation, and not about the diagnosis of MUPS. As stated in the background section this provides only a comparison on this topic.

### Implications for practice

The management of sick-listed employees with severe MUPS is hampered by low recognition by the OHP. For the employee it is important that this morbidity is managed. This can be improved by education and training of the OHP, and by liaison-psychiatric consultation with the employee. In the occupational health setting self-report questionnaires such as the PHQ and the 4DSQ may be useful instruments for the diagnosis of severe MUPS.

Adequate treatment of severe MUPS and adherence to management guidelines reduce medical consumption, improve physical functioning and may lead to symptom reduction [44-46]. If symptoms persists, the most appropriate evidence-based interventions, according to reviews [47,48], are cognitive behavioral treatment and antidepressant medication. For persistent health anxiety, cognitive behavioral therapy has been found to be effective [49].

Our results show that employees with severe MUPS are at least partly open to other than somatic explanations for their physical complaints. Training the OHP to explore the symptom attributions made by the employees might be worthwhile because this could lead to more adequate explanation and reassurance [50].

### Implications for further research

It is important to monitor the scores of sick-listed employees with severe MUPS on the course of their complaints (as to the severity of MUPS, psychiatric morbidity and functional limitations) after return to work. Such a study may indicate whether they remain at risk for frequent sick leave and which determinants are responsible for these eventual recurrences.

Longitudinal research is needed to find out whether severe MUPS are a determinant for prolonged sick leave, as severe MUPS are associated with more functional limitations.

Research is also needed to investigate the process of how a working employee develops higher scores of MUPS, psychiatric morbidity, distress, and functional limitations, and as a result reports sick-listed.

## Conclusion

Severe MUPS are more prevalent in sick-listed employees than in the general working population. Severe MUPS are accompanied by higher levels of psychiatric co-morbidity, health anxiety and distress in sick-listed employees than in general practice patients.

Severe MUPS are associated with multiple functional impairments, but we found indications that it is insufficiently recognised by the OHP. We recommend that OHPs receive guidelines, education and training on this subject, and on the use of questionnaires as instruments to detect and monitor MUPS.

## Competing interests

The authors declare that they have no competing interests.

## Authors' contributions

RH, BK, NB en JWG contributed to the design. RH collected the data. PK carried out the data analysis and gave methodological advice. All authors participated in the data-interpretation. RH wrote he manuscript. All authors revised the manuscript for important intellectual content. All authors have read and approved the final manuscript.

## Pre-publication history

The pre-publication history for this paper can be accessed here:

http://www.biomedcentral.com/1471-2458/9/440/prepub
